# Elevation of D-dimer levels are associated with early need for mechanical ventilation support in patients with COVID-19

**DOI:** 10.1186/s12890-023-02551-z

**Published:** 2023-08-03

**Authors:** Asmaa Ali, Wu Liang, Ahmed Samir Abdelhafiz, Mai M. Saleh, Heba Salem, Eman M. Moazen, Maram I. Elmazny, Mohammed Abdallah Rakha, Seham Ezzat Fathy Elfeky

**Affiliations:** 1 Department of laboratory medicine, School of Medicine, Jiangsu University, Zhenjiang, 212013 China; 2 Department of pulmonary medicine, Abbassia Chest Hospital, MOH, Cairo, Egypt; 3 Department of respiratory allergy, Al-Rashed Allergy Centre, MOH, Kuwait, Kuwait; 4 Department of Clinical Pathology, National Cancer Institute, Cairo University, Kasr Al-Aini Street, from El-Khalig Square, Cairo, 11796 Egypt; 5 Department of chest disease, Faculty of Medicine, Tanta University, Tanta, Egypt; 6 Department of chest disease, Faculty of Medicine, Al-Azhar University, Cairo, Egypt; 7 Department of anesthesia, intensive care and pain, Faculty of Medicine, Tanta University, Tanta, Egypt

**Keywords:** Severe COVID-19, Invasive mechanical ventilation, D-dimer

## Abstract

**Background:**

Severe COVID-19 disease is typically associated with an urgent need for supplemental oxygen therapy that may be successfully delivered through conventional methods or require invasive mechanical ventilation. Early prediction of the need for invasive mechanical ventilation could significantly improve outcomes of COVID-19 patients. Plasma levels of D-dimer and a number of inflammatory markers as well as values of complete blood counts, all measured in the first two days of hospital admission of COVID-19 patients, were evaluated for their significance as predictors of the eventual need for invasive mechanical ventilation support as well as their values as predictors of post-ventilation morbidly and mortality.

**Methods:**

This retrospective cohort study was conducted at a single center and included data pertaining to 200 patients with previously confirmed moderate to severe COVID-19 disease in the period between May 2021 and the end of December 2022. Data were retrieved from medical records for further analysis.

**Results:**

The mean (SD) age of patients stood at 59 (14) years of age, and with a majority of patients being male (77%). About 18% of cases, all of significantly older age, had been connected to invasive mechanical ventilation (IMV). Total leucocytic count (TLC), as well as levels of urea, creatinine, D-dimer, ferritin, and CRP in IMV patients were significantly higher than non-ventilated patients (*p* < 0.01 for all). In contrast, lymphocytic count, hemoglobin level, and platelet count were significantly lower in IMV patients (*p* < 0.001, 0.04, and 0.002, respectively). The mortality rate was significantly higher in IMV patients (*p* < 0.001). D-dimer independently predicted IMV demand (OR = 1, *p* = 0.001 in adjusted and unadjusted models). The utility of D-dimer was excellent; and the cutoff level of above 1415 µ/L showed sensitivity and specificity of about 92% and 76%, respectively. Also, the D-dimer level was very effective in predicting post-IMV survival; the AUC = 0.86, p = 0.02, and a cutoff value below 4558 µ/L was associated with 100% and 66% sensitivity and specificity, respectively.

**Conclusions:**

High D-dimer levels independently correlated with the need for invasive mechanical ventilation. Low levels of this marker could evidently predict post-IMV survival of mechanically ventilated COVID-19 patients. Measuring D-dimer levels during routine follow up of those patients would thus be useful in predicting patient outcomes.

## Introduction

The emergence and spread of the SARS-CoV-2 virus in Wuhan, China starting in December 2019 eventually led to the coronavirus disease (COVD-19) pandemic in early 2020. As the pandemic continued, concomitant studies led to a wealth of data that helped better clarify the pathogenesis of the disease, and improved treatment strategies. It emerged that one of the main pathogenic features of severe COVID-19 disease is the hypercoagulability state, characterized by prolonged prothrombin time and high levels of fibrinogen and fibrin degradation products such as D-dimer [1–3]. The activation of the coagulation cascades in COVID-19 patients, especially in critically ill patients, significantly increases the morbidity and mortality of this group of patients [4–6].

Severe COVID-19 disease is characterized by silent hypoxia. Ventilation-perfusion mismatching leads to reduced gas exchange. Accordingly, high-flow oxygen supply has become an obligatory part of any treatment plan [7–9]. COVID-19 causes hypoxia through a number of mechanisms, one of which is lung injury, typically associated with increased blood viscosity and thrombogenesis [10]. The hyper-coagulation state augments the hypoxic state of COVID-19 patients. Additionally, up-regulation of *FOXP3* occurs in severe COVID-19 disease [11]. Through its effect on the hypoxia-inducible factors (HIF), *FOXP3* indirectly causes coagulation in association with the pro-inflammatory cytokines TNFα and IL-1 (12–13). In addition, it has been reported that COVID-19 is associated with cardiac injury, which occurs due to a number of mechanisms. Cardiac injury could further complicate the state of hypoxia in these patients [14]. Hypoxia is associated to the development of dyspnea, which could persist even after recovery from COVID-19 [15].

With deterioration of oxygenation, when should invasive mechanical ventilation be a clinically wise decision? To date, the best timing for bronchial intubation is still unclear. In the current study we investigated the utility of different blood cells and coagulation markers as predictors of the need for invasive mechanical ventilation and scrutinized possible correlations between their levels and patient survival.

## Methods

### Patients and study design

This retrospective study included records of all patients above 18 years of age with confirmed COVID-19 pneumonia. Diagnoses had been confirmed using real-time polymerase chain reaction (RT-PCR) from nasopharyngeal swabs. Pneumonia had been confirmed through high-resolution computed tomography (HRCT), conducted at the Chest Department, Tanta University Hospital, Egypt, between May 2021 and the end of December 2022. Only patients with complete records were included in the study. All laboratory data were collected from medical files after approval from the Ethical Committee Board of the Faculty of Medicine, Tanta University.

Patients with severe COVID-19 pneumonia were categorized with respect to their oxygen demand during the first 48 h of admission into two groups: those who only needed conventional oxygen supply; and those whose treatment required invasive mechanical ventilation (IMV).

### Measurement and endpoint

The data collection sheet included (i) demographic characteristics of patients, including age and gender; (ii) associated comorbidities; and (iii) baseline laboratory data, with complete blood counts (CBC), results of liver and kidney function tests, levels inflammatory markers and levels of D-dimer especially diligently recorded. The chosen endpoints were in-hospital mortality as well as disease-associated complications.

### Statistical analysis

Data were collected in on a Microsoft Excel sheet and statistically analyzed using Sigma Plot for Windows version 12.5.0.38 (Systat Software, Inc, UK, 2011). The normality of data was measured using the Shapiro-Wilk test. Comparison between two means was performed using an independent t-test, and between two frequencies using a Chi-square test. Logistic regression analysis with adjusted and non-adjusted models was used to evaluate D-dimer as a predictor of early demand for IMV. Additionally, the receiver operating characteristics curve (ROC-curve) was used to estimate D-dimer’s predictive utility in relation to the need for IMV and post-IMV survival. An area under the curve (AUC) > 0.6 was deemed acceptable. Assuming an estimated D-dimer’s sensitivity of 95%, an alpha error of 0.05, a beta error of 0.1, and a COVID-19 prevalence of 85%, the minimum sample size required for patients who needed IMV stood at 19. Assuming an estimated specificity of 90%, the minimum sample size increased to 35; this minimum sample size provided 90% power. All tests were two-sided, and a p-value < 0.05 was considered significant.

## Results

### Patient characteristics

Initially, the authors reviewed data pertaining to 213 cases of confirmed COVID-19 infection. Thirteen ‘moderate’ cases were subsequently excluded. More than 2/3 of cases were male. The mean (SD) age at diagnosis stood at 59 (14) years of age. Those requiring IMV were significantly older (67.77 ± 14.9 versus 57.42 ± 13.11 years; *p* < 0.001) and/or had hypertension (52.78% versus 34.15%; *p* = 0.03). Moreover, patients in IMV group had significantly higher TLCs, urea, creatinine, D-dimer, ferritin, and CRP levels (p < 0.01 for all). On the other hand, lower lymphocytic counts, hemoglobin (HB) levels, and platelet (PLT) counts were significantly associated with the need for IMV support (*p* < 0.001, 0.04, and 0.002, respectively). Table 1 summarizes the basic characteristics of patients.

**Table 1:** Basic characteristics of COVID-19 patients (n = 110)

Variable	Total (n = 200)		Traditional Oxygen (n = 164)		Invasive MV (n = 36)		*p*
	**Mean**	**SD**	**Mean**	**SD**	**Mean**	**SD**	
Age (Year)	59.25	12.96	57.42	13.11	67.77	14.9	**< 0.001** ^**†**^
	**N**	**%**	**N**	**%**	**N**	**%**	
Male-sex	154	77	123	75	31	86.11	0.13ƪ
DM	77	37.5	63	38.41	14	38.89	0.91 ƪ
HTN	75	36.5	56	34.15	19	52.78	**0.03** ƪ
IHD	9	3.5	6	3.66	3	8.33	0.22 ƪ
	**Mean**	**SD**	**Mean**	**SD**	**Mean**	**SD**	
TLC (10^9^/L)	11.55	3.5	10.8	3.4	18.9	7.6	**< 0.001** ^**†**^
Lymphocyte (10^9^/L)	1.66	0.41	1.77	0.98	1.19	0.51	**< 0.001** ^**†**^
HB (mg/dL)	14.3	4.2	13.75	6.7	12.75	4.9	**0.04** ^**†**^
PLT (10^9^/L)	314.5	95	325	175.8	269.5	98.2	**0.002** ^**†**^
AST (IU/L)	47.5	13.2	39	11	82	32	**< 0.001** ^**†**^
ALT (IU/L)	59	21	55	29	67	27	**0.05** ^**†**^
Urea (mg/dL)	57	19	49	24	156.5	77.4	**< 0.001** ^**†**^
Creatinine (mg/dL)	1.2	0.4	1.1	0.4	1.8	0.7	**< 0.001** ^**†**^
D-dimer (µ/L)	956	242	733	360	6486	2710	**< 0.001** ^**†**^
Ferritin (ng/L)	986	233	779	210.5	2638	970.6	**< 0.001** ^**†**^
CRP (mg/dL)	99.4	22.6	78.7	19.2	234	93.7	**< 0.001** ^**†**^

Numerical data presented as mean and stander deviation, and categorical data as number and percentage, n: number, SD: stander deviation, IMV: invasive mechanical ventilation, DM: diabetes mellitus, HTN: hypertension, IHD: ischemic heart disease, TLC: total leukocyte count, HB: hemoglobin, PLT: platelets, AST: Aspartate transaminase, ALT: Alanine transaminase, CRP: C-reactive protein, †: independent t-test, ƪ: chi square test, *p* considered significant if ≤ 0.05

### Coagulopathy and need for mechanical ventilation support

#### Coagulopathy and need for mechanical ventilation support

Table 2 shows the value of blood cell counts, levels of inflammatory and coagulation markers in the prediction of the need for invasive mechanical ventilation. Every one unit increase in the TLC was associated with one-fold increase in the likelihood of IMV demand (OR = 1.18 and 1.16 in un-adjusted and adjusted models, p = 0.01 and 0.05, respectively). Conversely, every unit decrease in both lymphocytic and PLT counts were associated with a one-fold increase in the likelihood of IMV demand (OR = 1 and 0.99; *p* = 0.01; 0.5, 0.01, and 0.01, respectively). Moreover, increased levels of CRP and D-dimer independently predicted IMV demand (OR = 1 for all, and *p* = 0.02, 0.03, 0.001, and 0.001, respectively).

**Table 2:** Independent predictors for invasive ventilation demand

Factors	Coefficient	OR	95% CI	*P*
Un-adjusted				
TLC (10^9^/L)	0.17	1.18	(1.0359,1.3541)	**0.01**
Lymphocyte (10^9^/L)	0.00	1.00	(0.9978,0.9998)	**0.01**
HB (mg/dL)	-0.39	0.68	(0.4382,1.0449)	0.07
PLT (10^9^/L)	-0.01	0.99	(0.9868,0.9992)	**0.01**
Urea (mg/dL)	0.01	1.01	(1.0000,1.0267)	**0.04**
D-dimer (µ/L)	0.00	1.00	(1.0001,1.0006)	**0.001**
CRP (mg/dL)	0.01	1.01	(1.0005,1.0162)	**0.02**
Adjusted				
Age (Year)	-0.05	0.96	(0.8710,1.0492)	0.32
Male-Sex	-0.25	0.78	(0.0397,15.1616)	0.87
DM	0.93	2.54	(0.1848,34.8226)	0.48
HTN	0.44	1.56	(0.1665,14.5228)	0.70
IHD	0.60	1.82	(0.1171,28.3517)	0.67
TLC (10^9^/L)	0.15	1.16	(0.9888,1.3665)	**0.05**
Lymphocyte (10^9^/L)	0.00	1.00	(0.9973,1.0002)	**0.05**
HB (mg/dL)	-0.48	0.62	(0.3531,1.0805)	0.08
PLT (10^9^/L)	-0.01	0.99	(0.9769,1.0001)	**0.01**
AST (IU/L)	0.00	1.00	(0.9886,1.0100)	0.90
ALT (IU/L)	0.00	1.00	(0.9957,1.0077)	0.68
Urea (mg/dL)	0.02	1.02	(0.9960,1.0466)	0.07
D-dimer (µ/L)	0.00	1.00	(1.0002,1.0009)	**0.001**
CRP (mg/dL)	0.01	1.01	(0.9997,1.0254)	**0.03**

Test of fitness: Hosmer-Lemeshow X2 = 1.01, *p* = 0.99, OR: odd ratio, CI: Confidence interval, DM: diabetes mellitus, HTN: hypertension, IHD: ischemic heart disease, TLC: total leukocyte count, HB: hemoglobin, PLT: platelets, AST: Aspartate transaminase, ALT: Alanine transaminase, CRP: C-reactive protein, Test of significant: Logistic regression analysis with adjusted and un-adjusted models, *p* considered significant if ≤ 0.05

### Consequences of mechanical ventilation

As shown in in Table 3, the mortality rate was significantly higher in mechanically ventilated patients (81.25%), p < 0.001, and in acute respiratory distress syndrome (ARDS), pulmonary embolism, sepsis, and septic shock patients, p < 0.001 for all.

**Table 3:** Outcome of mechanical ventilation in the patients

Variable	Total (n = 200)		Traditional Oxygen (n = 164)		Invasive MV (n = 36)		P
	N	%	N	%	N	%	
Sepsis	20	10	8	4.88	12	33.33	**< 0.001** ^**†**^
Septic shock	10	5	2	1.22	9	25	**< 0.001** ^**†**^
ARDS	9	4.5	1	0.66	9	25	**< 0.001** ^**†**^
PE	6	3	2	1.22	4	11.11	**0.001** ^**†**^
Mortality	34	17	2	1.22	26	81.25	**< 0.001** ^**†**^
	Mean	SD	Mean	SD	Mean	SD	
LOS (days)	8	2.5	6	2	8	3.5	0.16^**†**^

Numerical data presented as mean and stander deviation, and categorical data as number and percentage, n: number, SD: stander deviation, IMV: invasive mechanical ventilation, ARDS: acute respiratory distress syndrome, PE: Pulmonary embolism, LOS: length of stay in hospital, †: independent t-test, ƪ: chi square test, *p* considered significant if ≤ 0.05

D-dimer level was a significant independent factor that predicted survival in IMV group; every one unit decrease in D-dimer level increased the likelihood of mechanical ventilation survival one time; OR = 1, *p* ≤ 0.05 in both adjusted and nu-adjusted models Table 4).

**Table 4:** Factors implicated in survival after IMV

Factors	Coefficient	OR	95% CI	P
Un-adjusted				
D-dimer	-0.01	1.00	(0.9992,1.0001)	**0.05**
Adjusted				
D-dimer	-0.01	1.00	(0.9992,1.0001)	**0.05**
Age	0.01	1.00	(0.9239,1.0833)	0.99
DM	-1.00	0.37	(0.0291,4.6706)	0.42

Test of fitness: Hosmer-Lemeshow X^2^ = 8.01, *p* = 0.43, OR: odd ratio, CI: Confidence interval, DM: diabetes mellitus, Test of significant: Logistic regression analysis with adjusted and un-adjusted models, *p* considered significant if ≤ 0.05

### The role of D-dimer in the management of COVID-19 patients

**Fig. 1:**
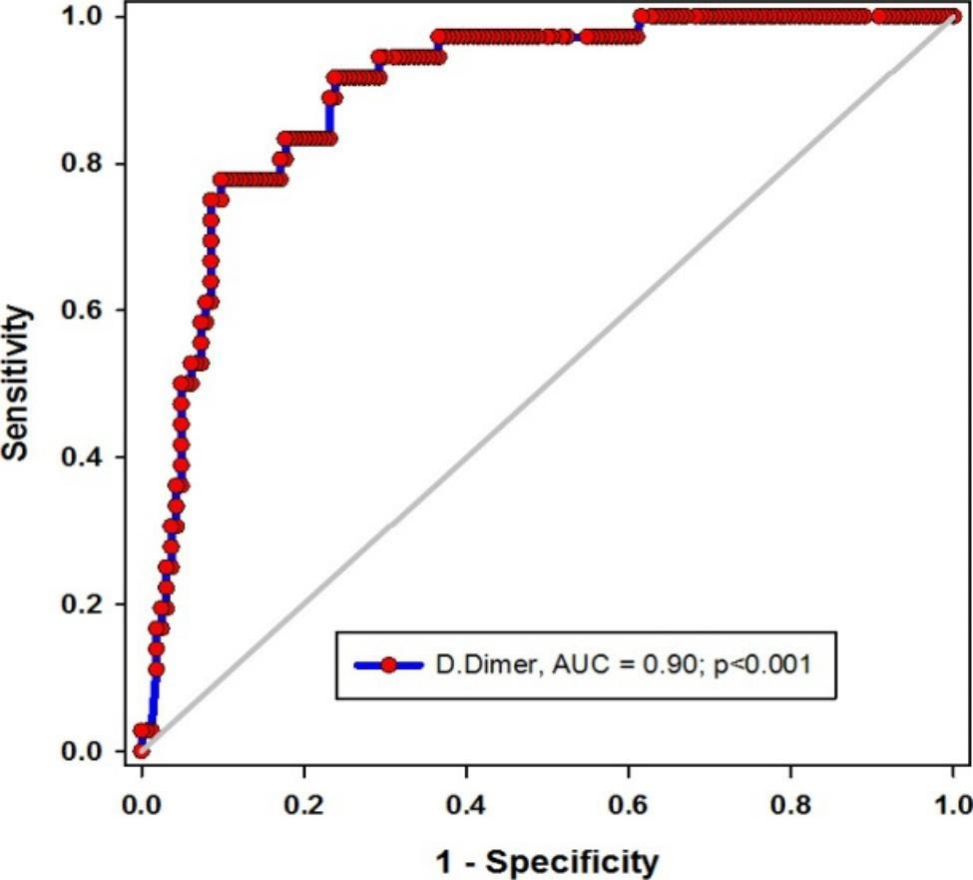
Utility of D-dimer in predicting IMV need *AUC: area under curve, p < 0.05 considered significant

**Fig. 2:**
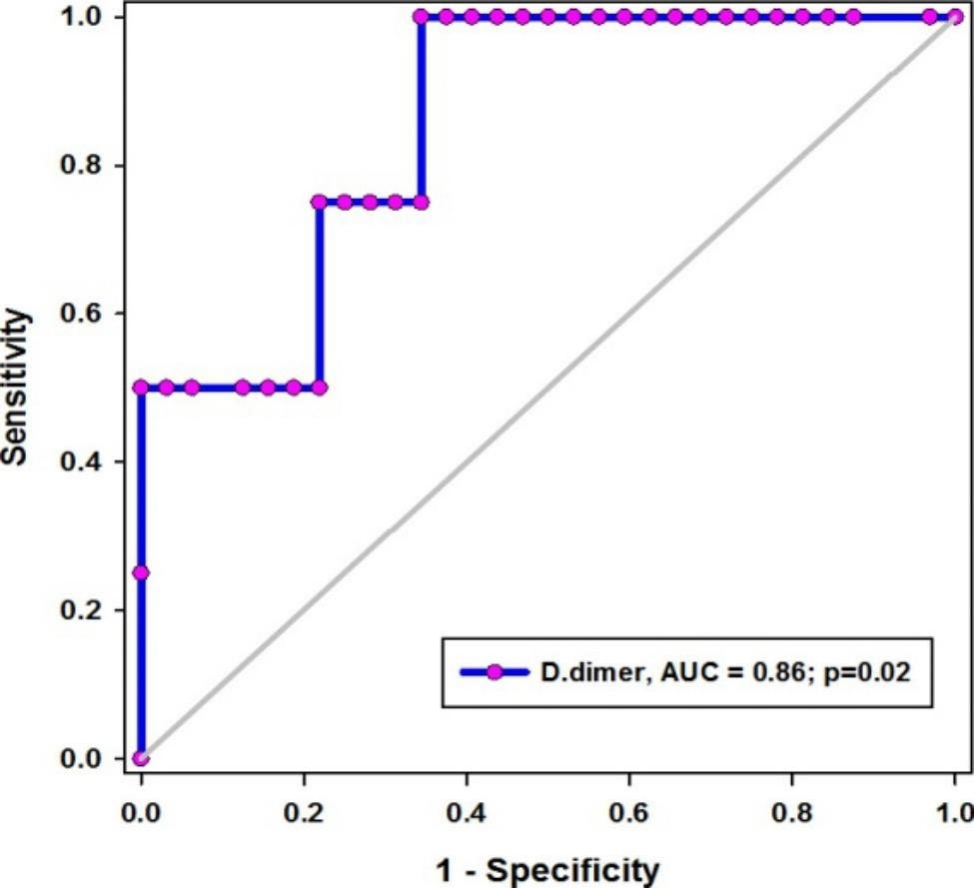
Utility of D-dimer in predicting MV survival *AUC: area under curve, p < 0.05 considered significant

The utility of D-dimer levels in was excellent in determining COVID-19 patients who needed IMV support (AUC = 0.9, *p* < 0.001) **(**Fig. 1**)**. The sensitivity and specificity of the cutoff value above 1415 µ/L were 92% and 76% respectively (Table 5). Additionally, D-dimer levels were exceedingly efficient in predicting post-IMV; AUC = 0.86, p = 0.02 **(**Fig. 2**)**. The sensitivity and specificity of the cutoff value below 4558 µ/L were 100% and 66% respectively (Table 5).

**Table 5:** Performance of D-dimer in COVID-19 patients

	D-dimer	Sensitivity	95% CI	Specificity	95% CI	PPV	NPV
IMV need	> 1415	92%	0.7753 to 0.9825	76%	0.6896 to 0.8251	46%	98%
IMV-survival	< 4558	100%	0.3976 to 1.000	66%	0.4681 to 0.8143	39%	100%

IMV: invasive mechanical ventilation, CI: confidence interval, PPV: positive predictive value, NPV: negative predictive value

## Discussion

Although the WHO has declared that COVID-19 no longer represents a public health emergency of international concern, their report highlighted that “SARS-CoV-2 has been and will continue to be circulating widely and evolving.” [16]. Understanding the pathophysiology of the disease, especially in severe cases, is crucial to the proper assessment of the need for any relevant intervention and the prediction of prognosis. The current study revealed that high levels of D-dimer were associated with a higher need for mechanical ventilation and poor survival of COVID-19 patients.

The mechanism by which SARS Cov-2 virus promotes thrombosis is still not well understood, and many theories have been proposed to explain this condition in COVID-19 patients [3, 17, 18]. One of the theories suggests an imbalance between inflammation and coagulation, where endothelial injury follows the activation of the innate immune system after viral invasion. In order to control this injury, activation of the micro-coagulation cascade takes place, leading to excess formation of thrombin and subsequent imbalance between thrombosis and fibrinolysis [3]. Peripheral blood components such as neutrophils and platelets, as well as a number of inflammatory markers including CRP, and a group of cytokines theoretically play a paramount role in this pathway [19, 20]. The hyper-inflammatory immune response, known as the *cytokine storm*, increases endothelial damage and subsequent thrombosis by releasing kinins from vascular smooth muscle cells. These kinins increase the permeability of blood vessels, leading to more inflammation and angioedema [21, 22]. The severe injury and thrombosis at the microvascular level leads to the development or exaggeration of hypoxia in COVID-19 patients [23]. Conversely, other studies have indicated that hypoxia could trigger thrombosis in moderate to severe COVID-19 [24]. Our results show that D-dimer acts as a marker of both hypoxia and thrombosis, allowing the prediction of the need for mechanical ventilation in severe cases of COVID-19.

Our results are comparable to those of a number of previous studies. A large study of ICU-admitted COVID-19 patients showed that D-dimer levels were associated with a higher risk of death [5]. Nemec et al. found that high levels of D-dimer at admission were associated with high intubation rates and poor prognosis of COVID-19 patients and recommended using this marker to predict the outcomes of these patients [25]. Similarly, Zhang et al. found that D-dimer admission levels could be used to predict mortality in these patients [26]. Their study found that a fourfold increase (D-dimer = 2.0 µg/ml) could be used as a cutoff to predict mortality in COVID-19 patients. A study by Yao et al. also found that D-dimer > 2.0 mg/L at admission was associated with disease severity and increased odds of mortality [27]. Our values were quite different, where a threefold increase was associated with increased odds of the need for mechanical ventilation, and about a nine-fold increase of D-dimer was associated with mortality in patients after mechanical ventilation. Our study group included only patients with pneumonia, which could explain the difference between the values in our study and the aforementioned studies.

Putting these data together, this study suggests that patients with higher levels of D-dimer are more likely to require early oxygenation, including IMV. D-dimer has traditionally been used as a marker of a patient’s coagulopathy state; and can be linked to the mechanism of hypoxia in patients with SARS-Cov-2 infection. As our data suggests, patients with D-dimer levels above 1415 u/L may benefit from elective IMV; the sensitivity and specificity of that cutoff number standing at 92% and 76%, respectively. Our data further suggests that, if the level of D-dimer did not exceed 4558 u/L, the possibility of IMV survival was higher. The sensitivity and specificity of the 4558 u/L cutoff point stood at 100 and 66%, respectively.

A number of treatment protocols including the Egyptian National Protocol have recommended that anticoagulation therapy be initially administered to COVID-19 patients with elevated D-dimer levels [28–33]. However, no recommendations have been made for elective invasive mechanical ventilation (IMV) for patients with severe COVID-19 pneumonia. Most decisions to initiate IMV rely on oxygen saturation levels measured through pulse oximetry or arterial blood gases. To address ventilation-perfusion mismatch, we considered early positive pressure ventilation in conjunction with anticoagulation therapy. Nevertheless, the question remained as to when to consider this approach. A flexible, easy marker such as D-dimer involved in developing hypoxia could be a helpful tool in predicting the need for invasive ventilation, as we have suggested as a treatment plan.

Long-term or post-COVID syndrome is the term that was coined to refer to symptoms that persist weeks or months after recovery from COVID-19 infection [34]. Patients with this syndrome suffer from a wide range of symptoms including fatigue, myalgia, dizziness, headache, dyspnea, and chest pain [34]. Understanding the exact mechanisms and finding biomarkers for this syndrome has been quite difficult. Patients may experience cardiac symptoms such as difficulty breathing or chest pain. However, these symptoms may not necessarily be due to cardiovascular involvement. It has been reported that dyspnea, and chest pain that occur in young adults as a part of this syndrome are unlikely signs of cardiovascular involvement [15].

In one of the studies, high baseline D-dimer levels were associated with inflammation but not a higher risk for venous thromboembolism [35]. These changes reportedly persisted for a period of time, with associated elevations of D-dimer levels persisting for up to 3 months after the infection, especially in patients who had suffered a severe form of the disease [36, 37]. This persistence in D-dimer levels raises questions about the possibility of use of D-dimer to understand and evaluate post-COVID syndrome.

### Conclusions and recommendations

Hypoxia and thrombosis are hallmarks of severe COVID-19, and each of them can exaggerate the other, adding to the magnitude of the disease. Our study proposes that D-dimer, commonly used as a marker of thrombosis, can also be used as a simple and inexpensive marker of hypoxia, to identify patients who need mechanical ventilation as early as possible. The study also suggests that D-dimer could also be used as a prognostic marker indicative of patient outcomes. The potential use of D-dimer for follow-up of the post-COVID syndrome should be further investigated, as these patients could experience long-term symptoms that affect their daily lives. Identification of ‘long COVID’ patients is crucial to their reception of the most optimal care needed.

This study has a number of limitations, being retrospective and having been conducted at a single center, restricting the number of cases analyzed. We recommend applying these recommendations in other centers to evaluate the potential use of D-dimer for the prediction of early need for IMV in COVID-19 patients.

## Data Availability

All data generated or analyzed during this study are included in this published article.
